# Editorial: Research in Sport Climbing

**DOI:** 10.3389/fpsyg.2021.752617

**Published:** 2021-09-22

**Authors:** Stefan Künzell, Jiri Balas, Vanesa España-Romero, David Giles, Pierre Legreneur

**Affiliations:** ^1^Institute for Sports Science, University of Augsburg, Augsburg, Germany; ^2^Faculty of Physical Education and Sport, Charles University, Prague, Czechia; ^3^Department of Teaching Physical Education, Fine Arts and Music, University of Cádiz, Cádiz, Spain; ^4^Lattice Training Ltd, Chesterfield, United Kingdom; ^5^Université de Lyon, LIBM, Lyon, France

**Keywords:** sport climbing, research, competitive sport, sport psychology, applied research

Sport climbing is enjoying growing popularity—on rock in the great outdoors and especially indoors on artificial climbing walls in inner-city gyms. Increased enthusiasm and participation in the sport has led also to greater interest in competitive climbing—ultimately leading to sport climbing being included as an Olympic discipline in Tokyo 2020 and beyond. It is hoped that the sport being showcased on the international stage will result in increased participation around the world.

Along with participation in climbing, interest in and research on the science of the sport has increased considerably. In 2011, the International Rock Climbing Research Association (IRCRA) was founded, which holds an international congress on research in climbing every 2 years. This Research Topic is the outgrowth of the last meeting of IRCRA in Chamonix in the summer of 2018, organized by its current president Pierre Legreneur.

Two strands with different objectives are emerging in climbing research. The first strand focuses on understanding and improving climbing performance. Performance-determining factors are sought and found, and a wide variety of training procedures are examined for their effectiveness. Conversely, the second strand focuses on climbers as a special population to be studied in terms of their perceptions, stress processing, and other personality traits. Such characteristics are examined to determine whether the practice of climbing promotes outcomes that are educationally or therapeutically desirable or just scientifically interesting. This Research Topic is predominately focused on the first strand.

Related to both the health and performance of climbing athletes, Gibson-Smith et al. assessed the dietary intake, body composition, and iron status. While the authors did not find significant differences between climbing ability groups (intermediate-advanced/elite-higher elite) for any of the parameters analyzed the results suggest experienced climbers are at risk of energy restriction and iron deficiency and monitoring of nutritional intake in training experienced athletes would be of benefit. Similarly, Joubert et al. surveyed a larger number of climbers using a web-based questionnaire and found that climbers are not immune to disordered eating, especially elite female climbers.

Concerning the technical movement performance, Reveret et al. analyzed the body motion of speed climbers (a sub discipline of climbing, involving the fastest possible ascent of a standardized 15 m route) in 3D. The ability to observe and quantify the velocity profile of speed climbers may help to highlight potential deviations from an optimal climbing path and shows the points where the upward movement stalls, providing coaches and climbers with actionable feedback on climbers speed performance.

Many athletes have to deal with stress and climbing is no different in this respect. For instance in bouldering competitions, a problem must be mastered in as few attempts as possible within a 4 or 5 min window, and the movements required often involve a high risk of failure. In their paper, Hill et al. presented a procedure to develop individual load-response profiles comparing difficulty with the number of attempts required, creating a system which may help coaches to find the optimal stress level in training for their athletes.

Considering the cognitive demands of the sport, Limonta et al. were interested in climber's route preview capacities and their ability to recall movement sequences. Analyzing both parameters in on-sight (without prior knowledge and practice of a route) and red-point (following practice) climbing the authors reported that, even for advanced climbers, the on-sight style is more physiological and psychological demanding than red-point climbing. The results highlight the interaction between perceptual, physiological, and psychological factors and the importance of psychological performance in on-sight climbing. Developing on the theme of the cognitive demand of on-sight climbing, Garrido-Palomino et al.'s study investigated the relationship between attention and self-reported climbing ability. The authors found that attention is positively related to on-sight but not red-point climbing ability, suggesting that higher level on-sight climbers are better able to attend to the task of climbing and not to external factors (e.g., risk of a fall) which may affect performance.

Research of the second strand is reported by Gajdošík et al. They studied climbers with different expertise as they climbed through an identical climbing route, one close to the ground and one at height. They were able to show that the perceived exertion for advanced climbers was in good agreement with the objective measurements. However, less proficient climbers overestimated their actual exercise intensity when climbing at height.

Fuss, Weizman et al. investigated the climbers abilities to perceive the roughness and grippiness of climbing holds. The construed holds from different materials that differed in these properties. They found that climbers mainly use grippiness for the evaluation of the holds. They use the estimated grippiness in planning the force they use to achieve the necessary friction efficiency and not slip off the hold.

In addition to the eight original research articles, the Research Topic is supplemented by three short reports. Sas-Nowosielski and Kandzia report the positive effect of post-activation potentiation on upper-body climbing-specific power exercise, Fuss, Tan et al. examine the behavior of heart rate during speed climbing, and Mitchell et al. examine the visual search strategies of experienced climbing coaches.

In anticipation of the approaching Olympic Games and climbing's debut, in this Research Topic, we have brought together research articles that predominately focus on improvements in the athletic performance of climbers. While developments in this area continue to be important and relevant the “second strand” of educationally and/or therapeutically relevant outcomes should be given more weight at future meetings and may become dominant content in a later Research Topic.

## Author Contributions

All authors listed have made a substantial, direct and intellectual contribution to the work, and approved it for publication.

## Conflict of Interest

DG was employed by company Lattice Training Ltd. The remaining authors declare that the research was conducted in the absence of any commercial or financial relationships that could be construed as a potential conflict of interest.

## Publisher's Note

All claims expressed in this article are solely those of the authors and do not necessarily represent those of their affiliated organizations, or those of the publisher, the editors and the reviewers. Any product that may be evaluated in this article, or claim that may be made by its manufacturer, is not guaranteed or endorsed by the publisher.

